# A hydrophobic residue in the TALE homeodomain of PBX1 promotes epithelial-to-mesenchymal transition of gastric carcinoma

**DOI:** 10.18632/oncotarget.17473

**Published:** 2017-04-27

**Authors:** Changyu He, Zhenqiang Wang, Li Zhang, Liyun Yang, Jianfang Li, Xuehua Chen, Jun Zhang, Qing Chang, Yingyan Yu, Bingya Liu, Zhenggang Zhu

**Affiliations:** ^1^ Shanghai Key Laboratory of Gastric Neoplasms, Department of Surgery, Shanghai Institute of Digestive Surgery, Ruijin Hospital, Shanghai Jiao Tong University School of Medicine, Shanghai, China; ^2^ Department of Otolaryngology, Ruijin Hospital, Shanghai Jiao Tong University School of Medicine, Shanghai, China; ^3^ Department of Clinical Oncology, Ruijin Hospital, Shanghai Jiao Tong University School of Medicine, Shanghai, China; ^4^ Clinical Research Center, Jiading District Central Hospital Affiliated Shanghai University of Medicine & Health Sciences, Shanghai, China

**Keywords:** PBX1, gastric carcinoma, epithelial-to-mesenchymal transition, tumorigenesis, hydrophobic binding

## Abstract

Pre-B-cell leukemia homeobox 1 (PBX1) was originally identified as a proto-oncogene in human leukemia. Although this protein has been shown to contribute to cellular development and tumorigenesis, the role of PBX1 in gastric carcinoma (GC) remains unclear. In this study, we observed increased expression of PBX1 in GC tissues compared with adjacent normal tissues. This increase in PBX1 expression levels negatively correlated with HOXB9 mRNA expression and was also associated with malignancy and metastasis. PBX1 promoted proliferation and metastasis of GC cells both *in vitro* and *in vivo*. These phenomena were also accompanied by epithelial-to-mesenchymal transition (EMT). Additionally, we observed that PBX1 promotes the expression of tumor growth and angiogenic factors. A structural model of the PBX1-HOX complex revealed that hydrophobic binding between PBX1 and the hexapeptide motif might be required for EMT induction. This analysis also demonstrated that the Phe^252^ residue in the first helix of the TALE homeodomain is involved in the latter hydrophobic binding reaction. *In vitro* data from PBX1 mutants suggest that PBX1 cannot promote tumorigenesis of GC cells *via* EMT induction when Phe^252^ residues lose hydrophobicity. It is likely that the presence of this residue is essential in facilitating hydrophobic binding with the hexapeptide motif. These findings suggest that PBX1 may be a potential target for GC treatment and this study provides a platform to elucidate the molecular mechanisms that underpin the role of PBX1 in GC tumorigenesis.

## INTRODUCTION

Pre-B-cell leukemia homeobox (PBX) was first identified in human preB cell acute lymphoblastic leukemia with t (1:19) chromosomal translocations [1, 2]. Additional studies have shown that PBX is an atypical homeodomain protein that can bind with the typical homeobox protein (HOX) and specific DNA strands during vertebrate embryogenesis and development [3]. PBX mutants can reverse HOX “loss-of-function” phenotypes during development; this result demonstrates that PBX is a cofactor of the HOX hexapeptide [4]. There are four members in the PBX family. PBX1, itself, is a product of a proto-oncogene, which suggests a role in tumorigenesis, metastasis, and chemoresistance in various cancers. Indeed, a previous study showed that increased expression of PBX1 promotes proliferation of melanoma and breast cancer cells [5–8]. These proliferative effects were dependent on the PBX1/HOX interaction. Furthermore, PBX1, in conjunction with PREP1, can induce epithelial to mesenchymal transitions (EMT) and lung cancer metastasis by increasing the TGF-β-induced SMAD3 nuclear signal [9]. A recent study has also revealed that chemoresistance in ovarian cancer is dependent on PBX1-dependent stem cell reprogramming [10].

PBX1 and its cofactors regulate transcription-specific activities during development and tumorigenesis [11]. The three-amino-acid loop extension (TALE) homeodomain of PBX1 binds specific DNA strands cooperatively with the hexapeptide HOX to increase promoter activation selectivity [12]. A family of conserved regions including the PBC-A and PBC-B domains are located in the N-terminus of the TALE homeodomain [13]. The fact that an E2A-PBX1 fusion protein is capable of inducing leukemia suggests that these conserved regions may be important during the regulation of tumorigenesis [14]. The HR domains in PREP and MEIS, which are sub-families belonging to the MEINOX family, are highly similar [15]. The PBC-A and PBC-B domains of PBX1 can directly bind with the HR domains [16], and competitive binding of PREP and MEIS with PBX1 determines whether tumorigenesis occurs [17]. In this study, in order to analyze the importance of the N-terminal region of PBX1, we developed a mutant cell line, ΔN140, missing the PBC-A domain. This allowed us to determine the importance of the PBX1 N-terminal region in GC tumorigenesis.

In previous work, we observed that HOXB9 hexapeptide mutations, which can disrupt PBX1/HOXB9 interactions, suppressed malignant GC phenotypes by inducing mesenchymal to epithelial transitions (MET). This occurrence suggests that PBX1 may be critical in the development of GC [18]. Nevertheless, the molecular mechanisms that underpin the PBX1-mediation of GC tumorigenesis remain unclear. Thus, this study aimed to elucidate the mechanisms that facilitate this phenomenon. As part of this analysis, we reported that PBX1 expression was increased in GC; we also observed that over-expression of PBX1 promoted proliferation, migration, and invasion of GC cells *via* EMT induction. These effects were dependent on the hydrophobic residue, Phe^252^, in the hexapeptide binding pocket of the TALE homeodomain.

## RESULTS

### PBX1 expression in GC

**Figure 1:**
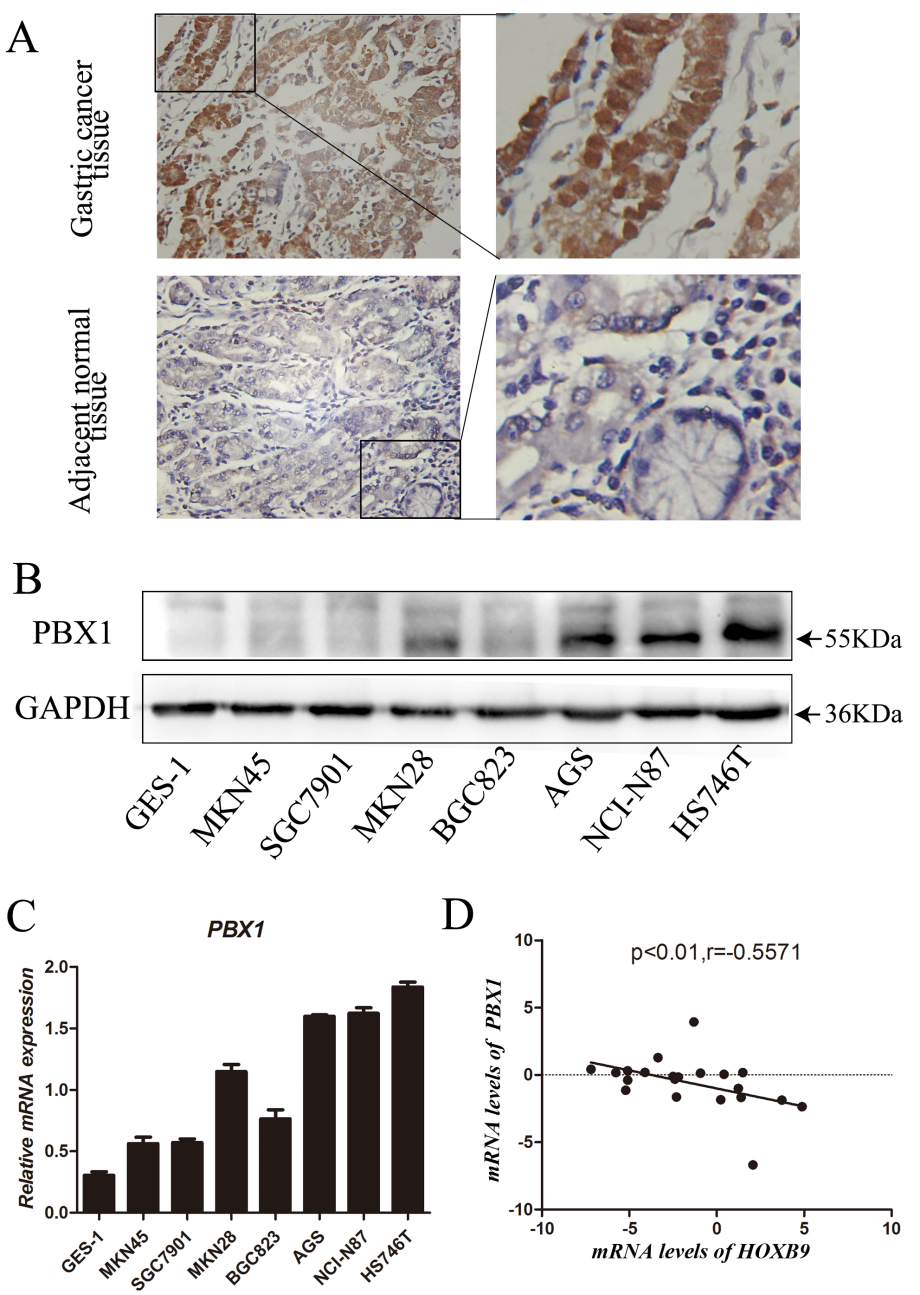
Expression of PBX1 in GC tissues and cells **A**., PBX1 protein expression in GC tissues and adjacent normal tissues was detected by immunohistochemistry staining. Brown staining indicates the presence of PBX1. PBX1 was mainly enriched in the nucleus and partially present in the cytoplasm of cells. PBX1 was highly expressed in GC tissues, while PBX1 was weakly expressed in adjacent normal tissues. **B**., PBX1 protein expression in seven different GC cell lines and the normal gastric mucosal epithelial cell line (GES-1), was examined by western blot analysis. GADPH expression was used to normalize the samples. **C**., The mRNA expression of *PBX1* in GC cells and normal gastric mucosa epithelial cells was examined using a qPCR assay. Seven different GC cell lines and the normal gastric mucosal epithelial cell line, GES-1, were used in this analysis. **D**., *PBX1* and *HOXB9* mRNA levels in 21 human GC tissues and correlation between *PBX1* and *HOXB9* mRNA levels.

PBX1 protein expression in GC and adjacent normal tissues was assessed using IHC (immunohistochemistry). The resultant data revealed that PBX1 exhibited increased expression in GC tissues compared with adjacent normal tissues. PBX1 was predominantly present in the nuclei, and partially expressed in the cytoplasm of cancer cells (Figure 1A). Statistical analysis of PBX1 expression and clinicopathological characteristics of 82 GC patients suggested that three clinical features were significantly correlated with PBX1 expression (Table 1). PBX1 expression was increased in GC tissues relative to that in the normal tissues (*p* = 0.008). Furthermore, it was observed that increased PBX1 expression in GC significantly correlated with a poor histological grade (*p* = 0.041) and lymph node metastasis (*p* = 0.034).

**Table 1:** Relationship between PBX1 expression and clinicopathology in 82 GC subjects

Features	Counts of Patients	IHC of PBX1		*p* value
		Weak	Strong	
**Tissues**				0.008*
Pericarcinoma	82	54	28	
Carcinoma	82	37	45	
**Gender**				0.713
Male	65	30	35	
Female	17	7	10	
**Age (years)**				0.339
≥ 60	44	22	22	
< 60	38	15	23	
**Tumor size**				0.409
≥ 5 cm	38	19	19	
< 5 cm	44	18	26	
**Histologic grade**				0.041*
Poorly differentiated	60	23	37	
Well&moderately differentiated	22	14	8	
**TNM stage**				0.073
I/II	22	13	8	
III/IV	60	24	37	
**LN metastasis**				0.034*
Existence	66	26	40	
Negation	16	11	5	
**Metastasis**				0.965
M0	73	33	40	
M1	9	4	5	

Statistical significance was assessed by Pearson's Chi-square test, *p*<0.05 was emphasized with an asterisk. LN metastasis: lymph node metastasis. TNM stage: the TNM classification of malignant tumors.

Western blot analysis was used to measure PBX1 protein expression in seven GC cell lines and one normal gastric mucosal epithelial cell line (GES-1). Relatively poor PBX1 expression was observed in GES-1, MKN45, SGC7901, and BGC823, and increased expression was observed in the AGS, NCI-N87, and HS746T GC cell lines (Figure 1B). Real-time PCR data showed that the *PBX1* mRNA expression was higher in AGS, NCI-N87 and HS746T GC cell lines compared with GES-1 cells (Figure 1C). Moderate expression was observed in MKN45, SGC7901, MKN28, and BGC823 GC cell lines (Figure 1C).

In previous work, we hypothesized that PBX1 may suppress HOXB9 in GC [18]. Thus, in this study we evaluated the correlation between *PBX1* and *HOXB9* in 21 GC tissues and the resultant data indicated (Figure 1D) a negative correlation between mRNA levels of PBX1 and HOXB9 in GC (*r* = −0.56, *p < 0*.01).

### Increased expression of PBX1 causes increased proliferation and migration of GC *in vitro*

**Figure 2:**
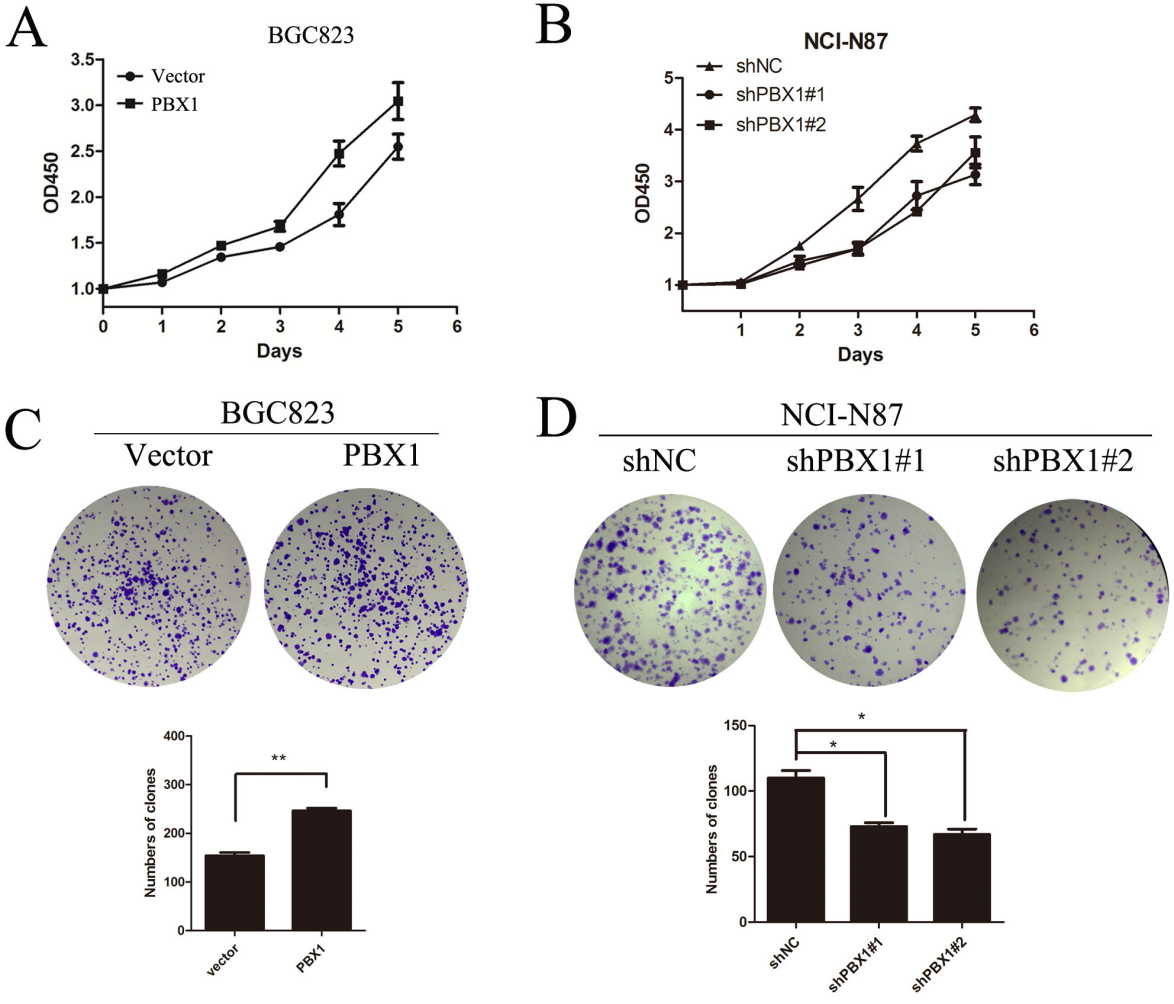
Effects of PBX1 on GC cell growth **A**. and **B**., Effects of PBX1 overexpression and knockdown on GC cell growth. Cell proliferation was measured using a water-soluble tetrazolium salt assay. This assay required Optical Density determination at a wavelength of 450nm. **C**., Effects of PBX1 overexpression on anchorage-independent BGC823 cell growth. Anchorage-independent growth assays were performed in 6-well plates and a total of ~1.0 × 10^3^ cells were seeded per well. Visible colonies with diameters exceeding 50 μm were counted. **D**., Effects of PBX1 knockdown on anchorage-independent NCI-N87 growth (plate colony formation assay). Anchorage-independent growth assays were performed in 6-well plates and a total of ~1.0 × 10^3^ cells were seeded per well. Visible colonies with diameters exceeding 50 μm were counted. Data are represented as means ± SD of five independent experiments (*p < 0.05, **p < 0.01).

To test whether *PBX1* acts as an oncogene in GC, we successfully constructed PBX1-transfected BGC823 cells and PBX1 knockdown NCI-N87 cells. BGC823 cells were used as a “gain-of-function” cell model because endogenous expression of PBX1 is poor, with related phenotypes being difficult to identify. NCI-N87 cells were used as a “loss-of-function” model and this cell line has previously been demonstrated to represent an effective model for the loss of PBX1 [19]. The genotype of both cell lines was confirmed by western blot analysis (Figure 5A and 5B) and qPCR (Figure 8A and 8C). It was observed that PBX1 overexpression promoted GC cell proliferation (Figure 2A), and suppression of PBX1 expression significantly inhibited GC cell proliferation (Figure 2B). A colony formation assay was subsequently performed and indicated that PBX1 overexpression in BGC823 cells increased colony formation compared with cells transfected with the pLVX-IRES-mCherry vector (*p < 0*.05, Figure 2C). PBX1 suppression in NCI-N87 cells resulted in a dramatic reduction in GC colony formation (*p <* 0.05, Figure 2D). Thus, these data indicate that PBX1 can modulate proliferation of GC cells *in vitro*.

**Figure 3:**
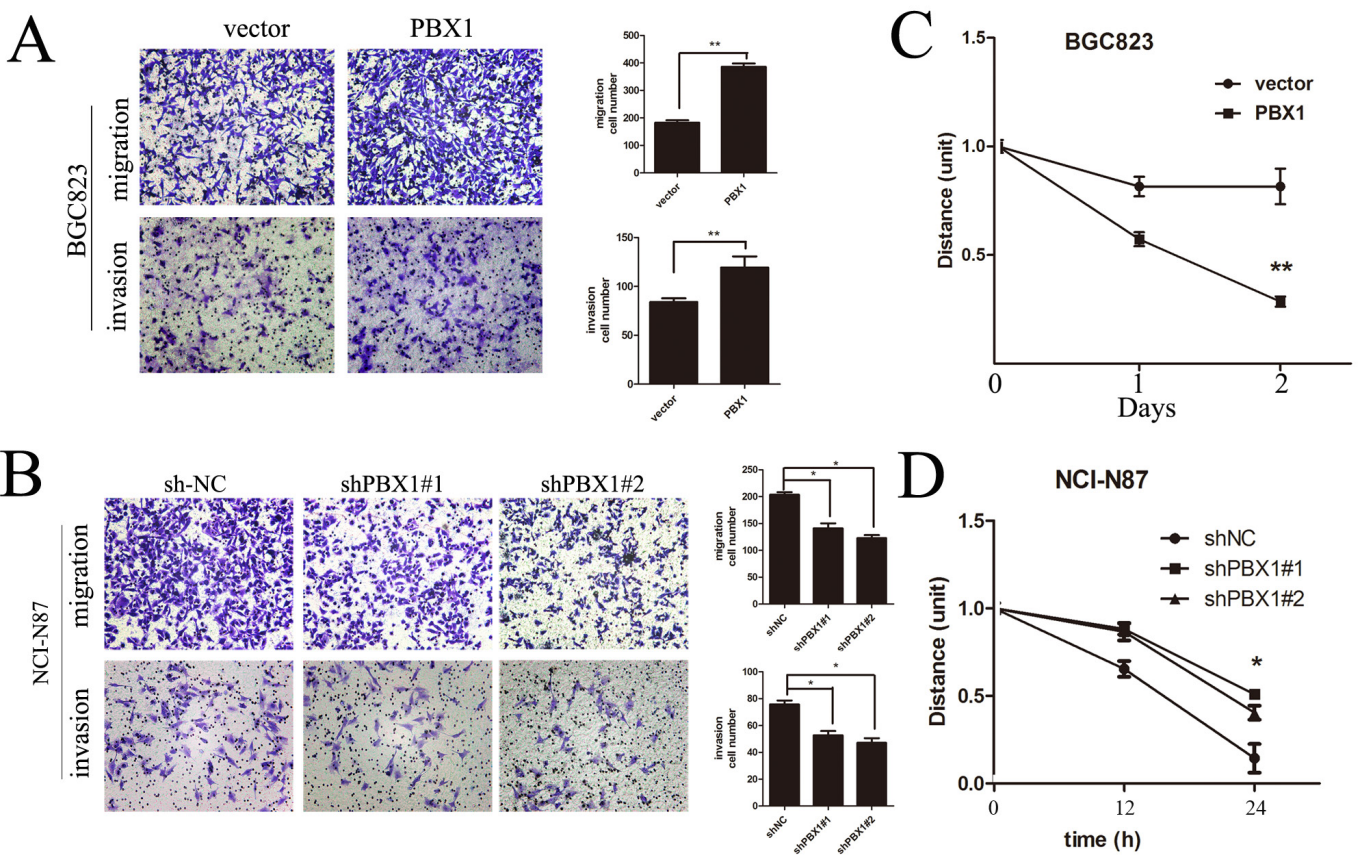
Effects of PBX1 on migration and invasion in human GC cells **A**., Representative images of migrated/invaded BGC823 cells through the chamber membrane. Mean migrated/invaded cells in BGC823/vector and BGC823/PBX1 groups. **B**., Representative images of migrated/invaded NCI-N87 cells through the chamber membrane. Mean migrated/invaded cells in NCI-N87/shNC, NCI-N87/shPBX1#1 and NCI-N87/shPBX1#2 groups. Cells were counted in five randomly selected microscopic fields (*p < 0.05). Data are means ± SD of three independent experiments. **C**., Wound healing cell migration assays of BGC823/vector and BGC823/PBX1 cells. **D**., Wound healing cell migration assays of NCI-N87/shNC, shPbx1#1 and shPbx1#2 cells.

**Figure 4:**
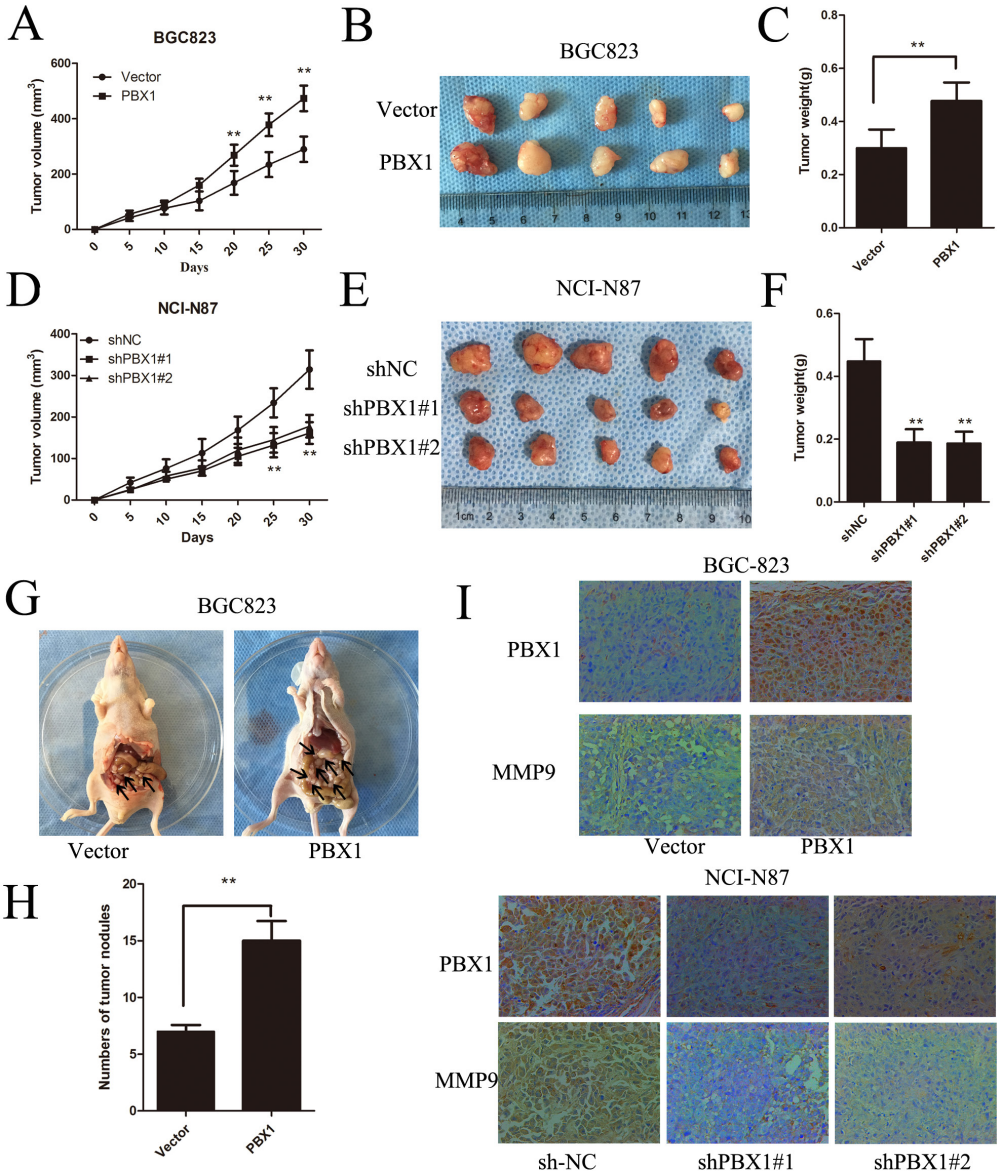
PBX1 promotes GC tumor growth and peritoneal metastasis *in vivo* **A**. and **D**., Growth kinetics of tumors after injection of BGC823/vector, BGC823/PBX1, NCI-N87/shNC and NCI-N87/PBX1 shRNA cells. Tumor volume was calculated using the Tumor volume equation where Tumor volume (V) = 1/2 × L × W^2^. **B**. and **E**., Representative tumor images from BGC823 cells and NCI-N87 cells. **C**. and **F**., Average tumor weights from nude mice. (*p < 0.05, **p < 0.01; *n* = 5 per group). **G**., Peritoneal nodules after 30 days (*n* = 5, per group). **H**., Average peritoneal nodules from nude mice. Data are means ±SD, *p < 0.05. **I**., PBX1 and MMP9 protein expression in BGC823 and NCI-87 was detected by immunohistochemistry staining. Brown staining indicates the presence of PBX1 and MMP9.

**Figure 5:**
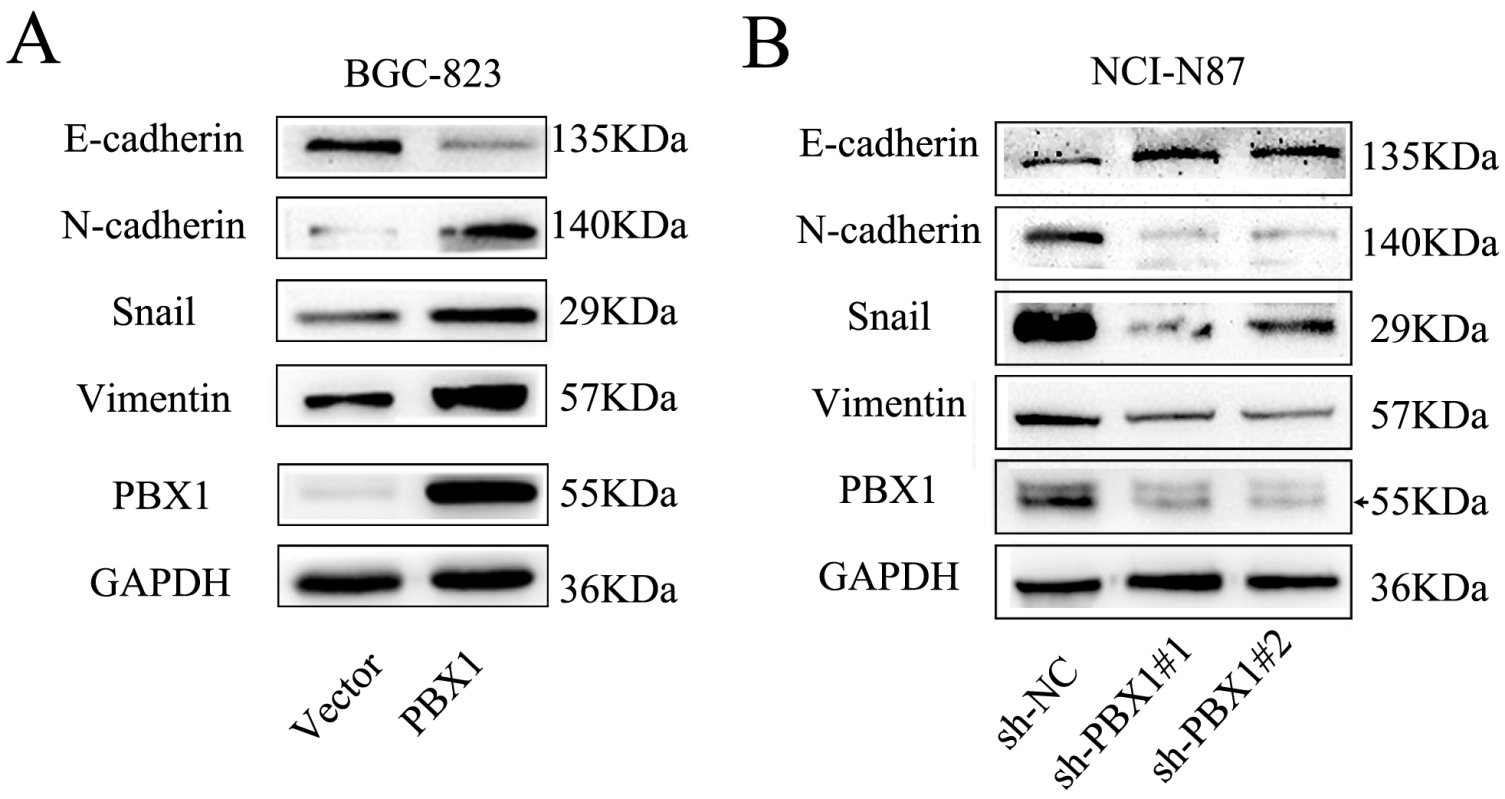
PBX1 enhanced EMT of GC cells **A**., Relative protein levels of EMT markers (E-cadherin, N-cadherin, Vimentin and Snail) in BGC823 cells. **B**., Relative protein levels of EMT markers (E-cadherin, N-cadherin, Vimentin and Snail) in NCI-N87 cells.

**Figure 6:**
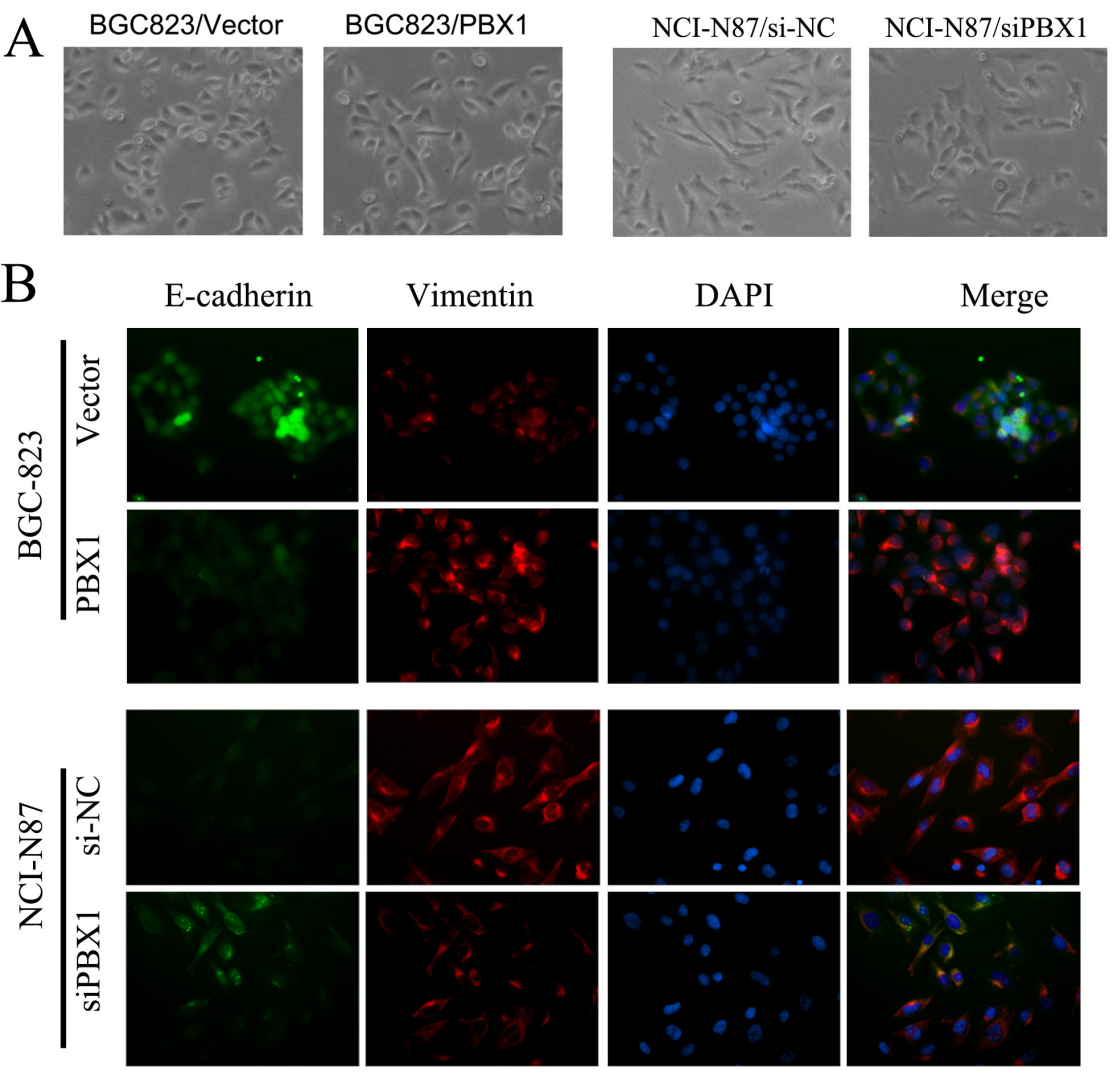
Effect of PBX1 overexpression and knockdown on cell morphology BGC823 cells transfected with PBX1 or control vectors, and NCI-87 cells transfected with siPBX1 or siNC. **A**., GC cells were observed under bright-field microscopy, with 200× original magnification utilized for all photomicrographs; **B**., Immunofluorescence analyses in BGC823 cells and NCI-N87 cells—E-cadherin (green), Vimentin (red), and DAPI (blue), with 200× original magnification utilized for all photomicrographs.

**Figure 7:**
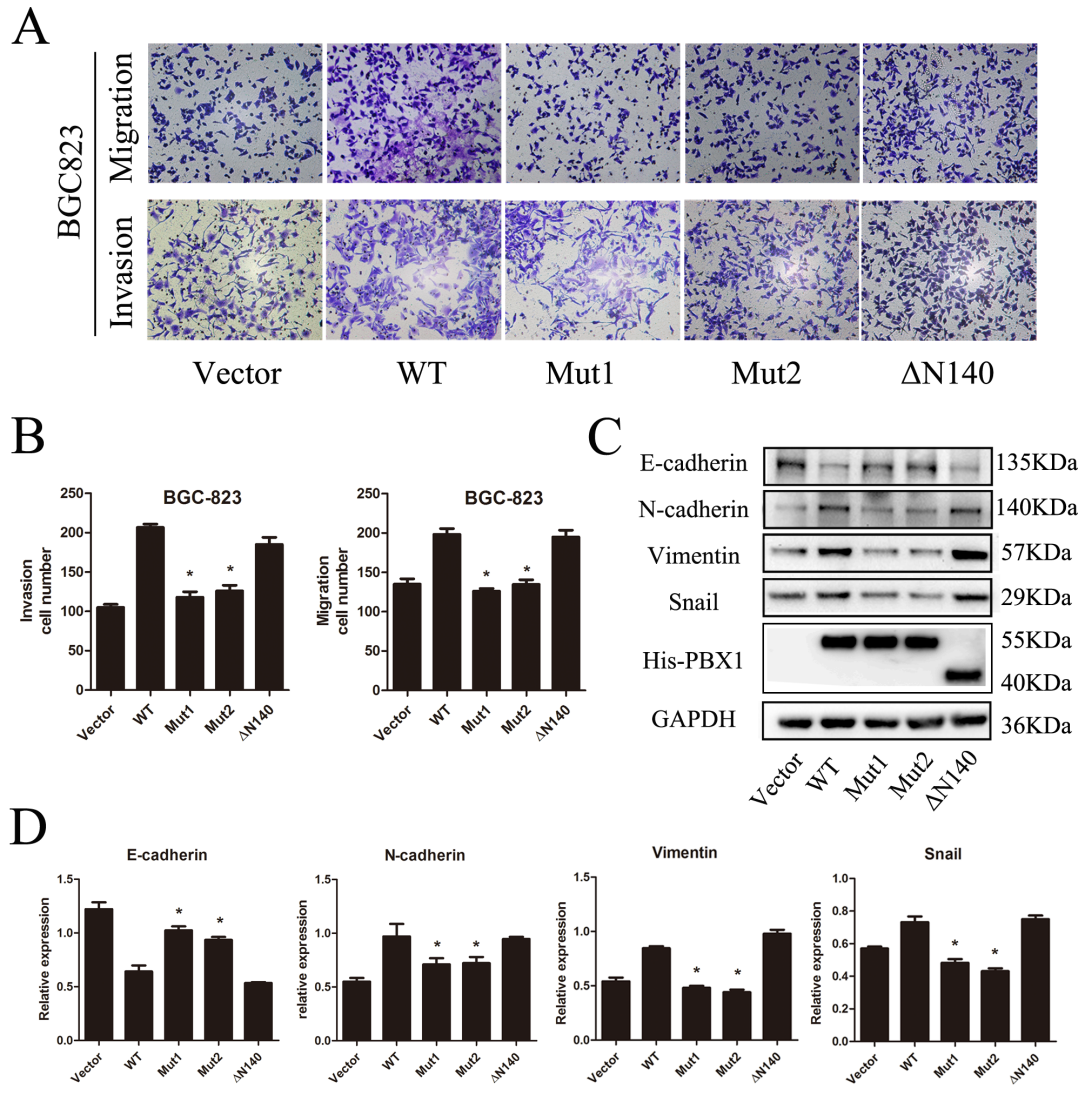
Analysis of potential regulatory sites in PBX1 **A**., Migration and invasion of BGC823 cells after transfection with WT, Mut1, Mut2 or ΔN140 PBX1 or non-targeting control. **B**., Quantification of GC cell migration and invasion after transfection with WT, Mut1, Mut2 or ΔN140 PBX1 or non-targeting control (*n* = 5, *p < 0.05). **C**. and **D**., E-cadherin, N-cadherin, Vimentin, and Snail protein expression measured using Western Blot analyses. The relative expression values were also tabulated for each of the analyzed proteins. Bars indicate standard errors (*n* = 5).

**Figure 8:**
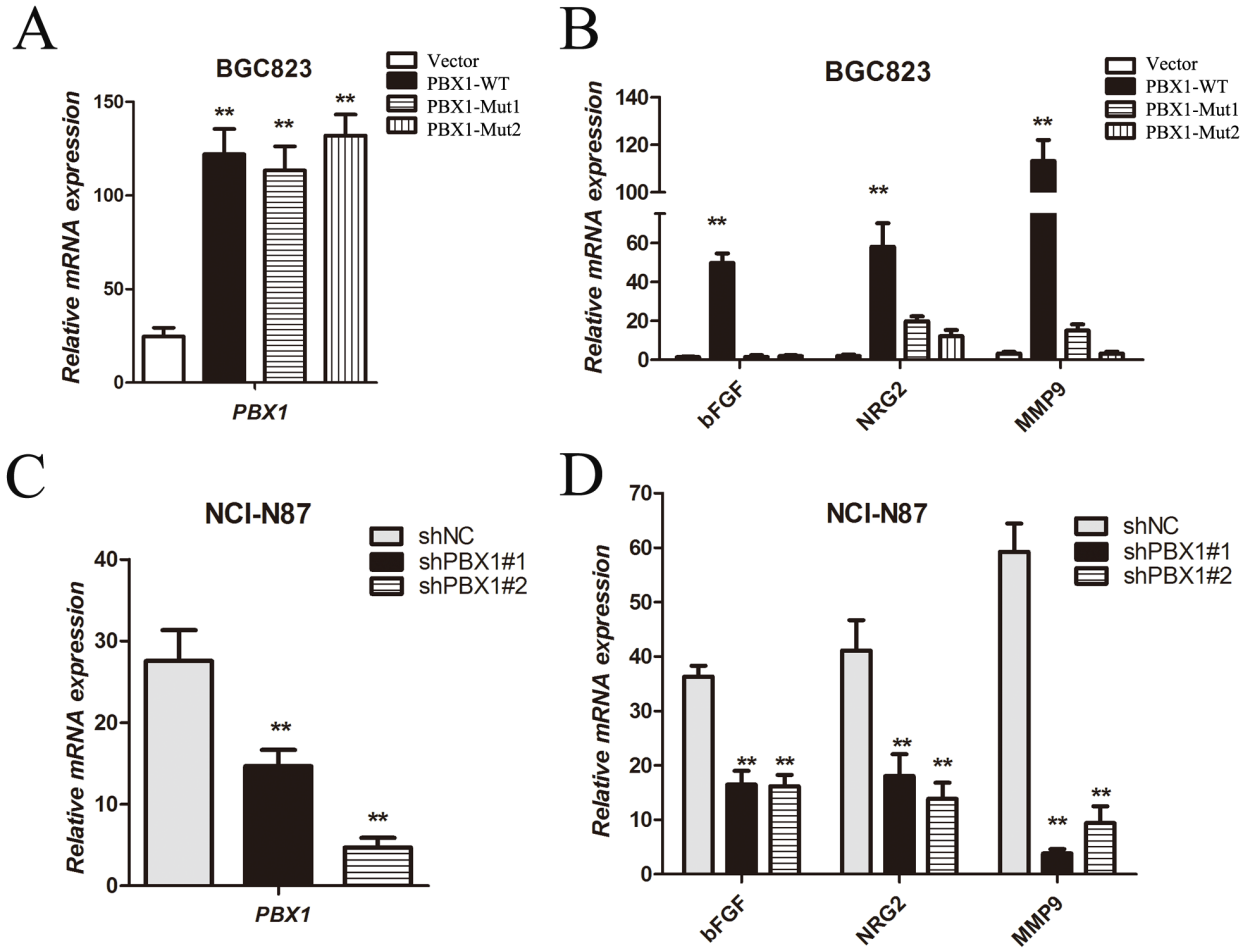
Tumor growth and angiogenic factor expression regulated by PBX1 in GC cells **A**. and **C**., Relative *PBX1* mRNA expression was quantified using real-time qPCR. This analysis was performed for vector, PBX1-WT, PBX1-Mut1, PBX1-Mut2, shNC, shPBX1#1 and shPBX1#2 groups. **B**. and **D**., Target gene *bFGF*, *NRG2*, and *MMP9* mRNA in cells was quantified using real-time qPCR. Data are means ±SD. Bars indicate standard errors (*n* = 5, *p < 0.05, **p < 0.01). This analysis was performed for vector, PBX1-WT, PBX1-Mut1, PBX1-Mut2, shNC, shPBX1#1 and shPBX1#2 groups.

To study *in vitro* migration and invasion of PBX1-transfected or knockdown GC cell lines, we used transwell migration and invasion assays and observed that PBX1 overexpression significantly increased BGC823 cell migration and invasion (*p < 0*.05, Figure 3A). Furthermore, knockdown of PBX1 expression suppressed migration and invasion of NCI-N87 cells (*p < 0*.05, Figure 3B). Following a wound healing cell migration assay, we observed a rapid closure in PBX1-transfected wounded cells by day 1 and the wound was virtually undetectable by day 2. For wounded cells transfected with the pLVX-IRES-mCherry vector, recovery was slower (Figure 3C). In contrast, suppression of PBX1 expression by shPbx1#1 or shPbx1#2 inhibited the closure of wounded cells (Figure 3D).

### PBX1 promotes tumor growth and peritoneal metastasis of GC *in vivo*

In nude mice experiments, PBX1-transfected BGC823 cells grew faster than those transfected with the pLVX-IRES-mCherry vector (Figure 4A), and tumor weights and volumes were greater for the PBX1 transfectants after one month (Figure 4B and 4C). There was also an increase in MMP9 expression along with a concomitant increase in PBX1 expression (Figure 4I). Suppression of PBX1 expression in NCI-N87 cells apparently inhibited GC growth in these mice (Figure 4D, 4E and 4F). This was coupled with a reduction in MMP9 expression (Figure 4I). Metastatic studies in nude mice indicated that PBX1-transfected BGC823 cells formed a greater number of nodules compared with controls (*p < 0*.01, Figure 4G and 4H). Thus, *in vivo* growth and metastasis of GC cells was promoted by PBX1, and these data agreed with *in vitro* results.

### PBX1 promotes tumorigenesis of GC through EMT induction

HOXB9 can suppress malignant phenotypes of GC cells through EMT suppression. Although PBX1 can bind with HOXB9, the role of PBX1 in GC remains unclear. Thus, we measured the expression of epithelial and mesenchymal markers and noted that PBX1 overexpression in BCG823 cells significantly decreased E-cadherin and increased N-cadherin, vimentin and Snail (Figure 5A). Knockdown of PBX1 expression in NCI-N87 cells increased E-cadherin expression and decreased N-cadherin, vimentin and Snail expression (Figure 5B).

Following morphological examination, we observed that BGC823 cells were clustered and displayed epithelial features following transfection with vector controls. The BGC823 cells exhibited a spindle-like shape, and the associated cells were scattered and detached from each other following transfection with PBX1 (Figure 6A). A reversal in cellular shape and arrangement was observed after knockdown of PBX1 expression by siPBX1 in NCI-N87 cells (Figure 6A, Supplementary Figure S1). To verify GC cellular morphology changes induced by PBX1, immunofluorescence assays were used to analyze E-cadherin pericellular staining and vimentin microfilaments. BGC823 cells transfected with the PBX1 vector displayed less green fluorescence (E-cadherin) and more red fluorescence (vimentin) compared with BGC823 cells transfected with the empty vector (Figure 6B). Knockdown of PBX1 expression in NCI-N87 cells increased E-cadherin expression but suppressed vimentin expression compared with the controls (Figure 6B). The cellular morphology alterations that were observed in GC cells suggested that PBX1 could promote tumorigenesis of GC cells *via* EMT induction.

### PBX1 potentially promotes tumorigenesis of GC *via* the hydrophobic residue, Phe^252^, in its TALE homeodomain

Amino acid sequence alignments of the PBX family are shown in Supplementary Figure S2A. These sequences display three conserved domains, PBC-A (Supplementary Figure S2A, red), PBC-B (Supplementary Figure S2A, purple) and the TALE homeodomains (Supplementary Figure S2A, green) [20]. Secondary structures of these conserved domains consist of four α-helixes, linked together by unstructured loops that vary in length. The N-terminus of the PBC-A domain and the C-terminus of the TALE domain contain flexible regions which are poorly conserved (not shown in Supplementary Figure S2A). We subsequently predicted the HOXB9 structure and simulated the interactions associated with the PBX1-HOXB9-DNA complex as previous described (Supplementary Figure S2B) [18]. Using this model, PBX1 binds to double-stranded DNA *via* the third α-helix of the TALE homeodomain [21, 22]. The hexapeptide HOX binds to double-stranded DNA and interacts with the TALE homeodomain *via* its hexapeptide motif [23]. In the TALE homeodomain, several residues were distributed in the first α-helix, the TALE loop, and the third α-helix. The presence of these residues created a small pocket for hexapeptide motif binding (Supplementary Figure S2A, light green). Only the benzyl side chain of Phe^252^ provides a potential hydrophobic surface for binding. Thus, binding between indole and benzyl side chains (Supplementary Figure S2B) may be essential for PBX1-dependent EMT induction of GC. In addition, there are six identifiable *PBX1* gene mutations in GC tissues in the *COSMIC* database (Supplementary Table 4). Among these mutations, five potentially affect the PBX1 amino acid sequence. These mutations are predominantly distributed in the PBC-A and PBC-B domains and can be competitively recognized by PREP and MEIS during tumorigenesis regulation.

To explore the regulatory sites of PBX1 during EMT induction, three PBX1 mutants were constructed. Phe^252^ of PBX1 was substituted with Alanine (Mut1) or Arginine (Mut2) and N-terminal residues (1-140, including the PBC-A domain) were deleted to generate the truncated ΔN140 mutant. Data from transwell migration and invasion assays showed that cell migratory and invasive capacities increased after WT and ΔN140 transfection compared with vector, Mut1 and Mut2 transfection. This suggests that up-regulation of PBX1 can no longer promote the migration and invasion of GC cells after Phe^252^ loses its hydrophobic characteristics (Figure 7A and 7B). ΔN140 PBX1 could promote migration and invasion of GC cells in a similar manner to WT PBX1, indicating that the PBC-A domain may not be a crucial regulatory site for PBX1 during EMT induction.

Epithelial and mesenchymal marker expression were measured using Western blot analysis (Figure 7C and 7D) and the resultant data showed that E-cadherin was suppressed in both WT and ΔN140 PBX1-transfected GC cells compared to GC cells transfected with control vectors. Mesenchymal markers (N-cadherin, vimentin, and Snail) were increased in GC cells overexpressing WT or ΔN140 PBX1 compared to controls, Mut1 or Mut2. Malignant phenotypic changes in GC cells as well as epithelial and mesenchymal marker expression changes demonstrate that the Phe^252^ hydrophobic residue in the TALE homeodomain is essential for PBX1-dependent EMT induction in GC. In addition, the PBC-A domain has no apparent regulatory effect during EMT.

### PBX1 increases the expression of tumor growth and angiogenic factors

Various tumor growth and angiogenic factors including VEGF (vascular endothelial growth factor), bFGF (basic fibroblast growth factor), TGF-β (transforming growth factor β) and NRG2 (neuregulin 2) are regulated by HOXB9 in a variety of cancers [18, 24]. Chemokines and MMPs (matrix metalloproteinases) can be released during EMT [25]. Although previous studies have shown that PBX1 can regulate the transcriptional activity of specific genes cooperatively with the HOX hexapeptide [12], the role of PBX1 in the regulation of tumor growth and angiogenic factors remains unclear. As part of this analysis, *bFGF*, *NRG2* and *MMP9* mRNA expression were measured and data showed that overexpression of WT PBX1 significantly increased expression of *bFGF*, *NRG2* and *MMP9* (Figure 8A and 8B). However, upon mutation of the hydrophobic Phe^252^ residue in the TALE homeodomain, a decrease in the expression of *bFGF*, *NRG2* and *MMP9* (to almost basal levels) in BGC823 cells was observed (Figure 8B). Two PBX1-specific antisense oligonucleotides, shPBX1#1 and shPBX1#2, significantly reduced expression of *PBX1* in NCI-N87 cells (Figure 8C). The expression of *bFGF*, *NRG2* and *MMP9* was significantly decreased following suppression of PBX1 expression (Figure 8D). Thus, tumor growth and angiogenic factor expression increased during PBX1-dependent EMT induction. Furthermore, the Phe^252^ residue in the TALE homeodomain of PBX1 is critical in the expression of tumor growth and angiogenic factors in GC cells.

## DISCUSSION

Although previous studies have reported that PBX1 can promote melanoma, breast cancer and lung cancer [5–8], the role that this protein plays in GC is unclear. In this study, we observed that the expression of PBX1 increases in some GC tissues compared with adjacent normal stomach tissues, suggesting that up-regulation of PBX1 might be an important factor in GC tumorigenesis. Additional *in vitro* and *in vivo* analyses suggest that *PBX1* can promote growth and metastasis of GC cells. Thus, it is speculated that *PBX1* may act as an *oncogene* in GC.

Increased PBX1 expression has been observed in adenocarcinomas associated with poorly differentiated tumors or lymph node metastasis, suggesting that increased PBX1 expression correlates with malignant GC pathologies. *HOXB9* mRNA expression is reduced in some *PBX1*-expressing GC tissues, and the occurrence of a moderately diminished relationship between *PBX1* and *HOXB9* expression indicates that PBX1 and HOXB9 may be considered tumor markers when attempting to determine GC malignancy. One limitation pertaining to this study is the fact that only HOXB9 was analyzed during this analysis. Several other HOX proteins are known to bind PBX1; however, the relationship between HOXB9 and PBX1 was the focus of this study.

PBX1 has also been reported to promote EMT induction in lung cancer [9]; however, its role in EMT during GC remains to be elucidated. In this study, over-expression of PBX1 resulted in a decrease in E-cadherin expression and an increase in N-cadherin, vimentin and Snail expression. Suppression of PBX1 expression reversed expression patterns associated with epithelial and mesenchymal markers. Molecular changes in GC cells stimulated cellular morphology changes. GC cells displayed a greater number of mesenchymal-like properties in PBX1-transfected BGC823 GC cells and a greater number of epithelial-like properties in PBX1 knockdown NCI-N87 GC cells. Previous studies have shown that tumor growth and angiogenic factors including *bFGF*, *NRG2* and *MMP9* promote EMT during tumorigenesis [26–28]. Furthermore, *PBX1* expression increases *bFGF*, *NRG2* and *MMP9* expression and suppression of *PBX1* expression facilitates a reduction in *bFGF*, *NRG2* and *MMP9* expression. This suggests that PBX1 induces EMT by increasing the expression of downstream tumor growth and angiogenic factors.

As part of this analysis, amino acid sequence alignments and PBX1 structural models were studied to explore potential regulatory sites in the protein that might be involved in tumorigenesis and EMT induction during GC. We observed that PBX protein sequences are highly conserved in the regions encoding for PBC-A, PBC-B, and TALE homeodomains. However, despite this sequence conservation, tumorigenesis activities relating to PBX proteins have previously been demonstrated to be diverse [29, 30]. Thus, the specific function of the different PBX1 proteins may depend on the relevant cofactors that are coopted during transcription [31]. A previous study demonstrated that PBX1 can bind with the hexapeptide motif through its TALE homeodomain [21], or with PREP/MEIS through its PBC-A domain [16]. Thus, the hexapeptide binding pocket residues or the PBC-A domain of PBX1 may be considered as potential regulatory sites. We constructed a mutant based on the PBC-A domain (ΔN140) and two mutants based on Phe^252^ (Mut1 and Mut2). The latter mutations were speculated to affect the hydrophobic interaction between PBX1 and the hexapeptide motif of HOX. Following transfection with PBX1 Mut1 or Mut2, neither tumorigenesis nor EMT of GC cells was promoted. In the hexapeptide motif, the Trp residue is conserved and involved in the PBX1-HOX-DNA interaction [21, 22]. The benzyl side chain of Phe^252^ in the hexapeptide binding pocket of PBX1 provides a hydrophobic surface that facilitates binding with the indole side chain of Trp. This hydrophobic interaction between PBX1 and the hexapeptide motif is essential for tumor growth and angiogenic factor expression, and thus is essential for PBX1-dependent tumorigenesis and EMT induction of GC. The side chain of Tyr^260^ is also in close proximity to the side chain of Trp^179^ in the hexapeptide motif (at 4.3 angstrom). Considering that the tyrosyl side chain of Tyr^260^ can bond with the major chain of Trp^179^ through a hydrogen bond, it is likely that the Tyr^260^-Trp^179^ interaction is mainly facilitated by this hydrogen bond. Although MEIS and PREP can bind competitively with PBX1 to determine the fate of tumorigenesis [17], there is no evidence to suggest that the PBX1 PBC-A domain is involved in PBX1-dependent EMT induction of GC cells.

Several small molecules and synthetic peptides can mimic the hexapeptide motif to competitively bind with the hexapeptide binding pocket of PBX1 and suppress tumorigenesis in various cancers [6, 32–34]. Since the hydrophobic interaction between PBX1 and the hexapeptide motif might be important in the suppression of GC cells (regardless of additional binding with various hexapeptide HOXs), the clinical application of these small molecules and synthetic peptides may represent a promising therapeutic strategy for the treatment of GC.

## MATERIALS AND METHODS

### Tissue microarray and immunohistochemistry

The present study was approved by the Ethics Committee of Shanghai Ruijin Hospital and written informed consent was obtained from all patients (*N* = 82) who underwent radical resection between 2010 and 2014. Tissue samples were pathologically studied and the corresponding non-tumor locations were at least 6 cm from the cancerous tissue. Correlations between the expression of PBX1 and clinicopathological features were analyzed using a chi-squared test.

For immunohistochemistry, tissues were treated according to published protocols and stained tissues were scored as previously described [18]. Briefly, dewaxed tissue sections were incubated with a PBX1-specific antibody [35] (Santa Cruz Biotechnology, CA, USA). Next, the sections were incubated with a horseradish peroxidase (HRP)-conjugated secondary antibody. The resultant sections were developed using diaminobenzidine (DAB) solution and counterstained with hematoxylin. Scoring was assessed based on the intensity of staining and the percentage of positive tumor cells as previously described [36]. The results were categorized as follows: Intensity scores: 0—negative staining, 1—weak staining, 2—moderate, and 3—strong staining. Percentage positive tumor cell scores: 0: < 5%, 1: 5-25%, 2: 25-50%, 3: 50-75%, and 4: ≥75% positive cells. PBX1 staining positivity was determined by the following formula: overall score = percentage score × intensity score. Overall scores of 0-4 indicated weak expression, whereas scores of 4-12 represented strong expression.

### Molecular cloning, cell culture and transfections

His-tagged wild type and mutant PBX1 sequences were cloned into a pLVX-IRES-mCherry vector according to published methods [18]. The primers were listed in Supplementary Table 3. Phe^252^ of PBX1 was substituted with Alanine (Mut1) or Arginine (Mut2). The region containing residues 1-140 in the N-terminus (including the PBC-A domain) of PBX1 was deleted to generate the truncated ΔN140 mutant. GC cell lines MKN-45, SGC-7901, MKN-28, BGC823, AGS, NCI-N87, HS746T and an immortalized normal gastric epithelial GES-1 were preserved in the Shanghai Digestive Surgery Institute (Shanghai, China). The associated cell lines were cultured in Dulbecco's modified eagle medium (DMEM) supplemented with 10% fetal bovine serum (FBS). The PBX1 cDNA ORF was cloned into the pLVX-IRES-mCherry plasmid and lentivirus was used as the gene delivery vector. The resultant lentivirus was incubated with cells for 72 hours and transfected cells were selected using puromycin (at a concentration of 1.5 μg/mL). The transfectants were confirmed *via* Western blot analysis. PBX1 shRNA or siRNA were designed by Genepharma Company (Shanghai, China) and transfections were conducted using Lipofectamine 2000 (Invitrogen, NY, USA). Sequences of shPBX1 and siPBX1 were listed in Supplementary Table 5.

### Cell proliferation assay

GC cells were seeded in 96-well plates (2 × 10^3^ cells/well) and the plates were subsequently incubated for 5 days. Cellular proliferation was measured using a water-soluble tetrazolium salt assay (Cell Counting Kit-8, Dojindo, Kumamoto, Japan) according to the manufacturer's protocol and experiments were performed in quintuplicate.

### Plate colony formation assay

Anchorage-independent growth assays were performed in 6-well plates and a total of ~1.0 × 10^3^ cells were seeded per well. The cells were cultured using DMEM supplemented with 10% FBS. After 14 days, cell colonies were fixed with methanol and stained with crystal violet. Visible colonies with diameters exceeding 50 μm were counted.

### Transwell cell migration and invasion assays

Migration and invasion assays were performed using the Boyden chamber technique as previously described [18]. For the migration assay, ~1.5 × 10^5^ cells in 100 μL of serum-free medium were placed in the upper chamber (Corning Costar, NY, USA), and 600 μL of the same medium with 10% FBS were placed in the lower chamber. After 24 h, migrated cells were fixed with methanol, stained with 0.5% crystal violet solution, and counted in five random fields (magnification: 100×). For the invasion assay, the same protocol was used; however, the upper chamber was precoated with diluted Matrigel (BD Bioscience, San Jose, CA).

### Wound healing assays

GC cells were cultured to 100% confluence as monolayers and scratched with a sterile 20 μL pipette tip. Cell migration was evaluated at different time intervals post-scraping under an inverted phase-contrast microscope (Olympus, Germany). Distances between cell wound edges were scored.

### Western blot and immunofluorescence assays

Western blot analyses were performed using previously published methods [37]. The cells were lysed with RIPA lysis buffer and the amount of total protein was quantified using the BCA Protein Assay Kit. Protein samples were loaded onto SDS-PAGE gels and then transferred onto polyvinylidene difluoride (PVDF) membranes. The membranes were blocked in PBS-T buffer containing 5% non-fat dry milk and hybridized with primary antibodies. The primary antibodies including the PBX1 antibody (Santa Cruz Biotechnology, CA, USA) [35], the GAPDH antibody (Proteintech, Rosemont, USA), E-cadherin, N-cadherin, Snail, Vimentin and His-tag antibodies (Cell Signaling Technology, Boston, MA, USA) were utilized according to the manufacturers’ protocols. Finally, membranes were incubated with HRP-conjugated secondary antibody. The protein bands were visualized using enhanced chemiluminescence (ECL). The PathScan EMT Duplex IF Kit (Cell Signaling Technology, Boston, MA, USA) was used to measure EMT marker (E-cadherin and vimentin [38]) expression.

### RNA isolation, reverse transcription and real time quantitative PCR (qPCR)

Total RNA from tissues or cells was isolated using a Trizol kit (Invitrogen, NY, USA). The RNA was subsequently reverse-transcribed using AMV RT kit (Invitrogen, NY, USA) according to the kit instructions. cDNA was amplified using 2 × SYBR Green PCR mix (QIAGEN, CA, USA) using the primers listed in Supplementary Table 1. A melting-curve analysis was performed to confirm the specificity of the amplified PCR products. GAPDH gene expression was used as an internal control for normalization. Experiments were repeated three times.

### *In vivo* tumorigenicity and metastasis assay

Animal experiments were carried out according to a protocol approved by the Institutional Animal Care and Use Committee (IACUC) at Shanghai Jiao Tong University, and experiments were conducted according to the guidelines of the Animal Research Ethics Board of Shanghai Jiao Tong University, Shanghai, China. Nude male mice (Institute of Zoology Chinese Academy of Sciences, 3 weeks-of-age) were housed in the Animal Laboratory Unit, Shanghai Ruijin Hospital. For each experiment, five mice were used per group. For the tumor xenograft model, cells (1 × 10^6^) were inoculated subcutaneously into right flanks. Tumor length (L) and width (W) were measured by caliper every 5 days and tumor volume was calculated using the tumor volume equation where tumor volume (V) = 1/2 × L × W^2^ [37]. Tumor volume was subsequently plotted as a function of time for growth curves. Animals were sacrificed one month after cell inoculation and the tumor specimens were collected, imaged, and weighed. For the peritoneal metastasis study, 100 μL of a cell suspension (1 × 10^6^ cells) was injected into the abdominal cavity of each mouse. After one month, mice were killed *via* the cervical dislocation method, and the metastatic masses were observed and nodules were counted.

### Statistical analysis

Statistical analyses were performed using SPSS 22.0 software. Results were summarized as means ± SD. The distributions of quantitative variables were tested. Continuous variables were compared between groups using an unpaired *t*-test and a paired *t*-test within each group. Categorical variables were compared using the Pearson *χ*2 test. *p < 0*.05 was deemed to be statistically significant.

### Sequence alignment and structure prediction of PBX family

The amino acid sequences of PBX family members were obtained from the NCBI, with accession numbers recorded in Supplementary Table 2. DIALIGN was used to perform multiple sequence alignments [39]. The amino acid sequence of PBX1 was submitted to the PSIPRED service for secondary structure prediction. The multiple sequence alignments were performed using ALINE software [40]. The catalogue of somatic mutations in cancer (COSMIC) database was used to analyze the *PBX1* gene mutations. The mutations and copy number variation (CNV) that permitted the identification of gastric carcinomas are summarized in Supplementary Table 4 [41]. The tertiary structure model of the PBX1/HOXB9/DNA complex was generated as previously described [18]. This model was required for structure/function prediction analyses and was generated based on the crystal structure for the HOXA9-PBX1-DNA complex. This simulation was feasible due to the sequence similarities between HOXA9 and HOXB9.

## SUPPLEMENTARY MATERIALS FIGURES AND TABLES




