# Supplementary material for: A hydrophobic residue in the TALE homeodomain of PBX1 promotes epithelial-to-mesenchymal transition of gastric carcinoma
Source: Oncotarget. 2017 Apr 27;8(29):46818–33. doi: 10.18632/oncotarget.17473 (PMC5564525; doi:10.18632/oncotarget.17473)
Supplement: Supplementary file 1 [file oncotarget-08-46818-s001.pdf]

A hydrophobic residue in the TALE homeodomain of PBX1 promotes epithelial-to-mesenchymal transition of gastric carcinoma

Supplementary Material

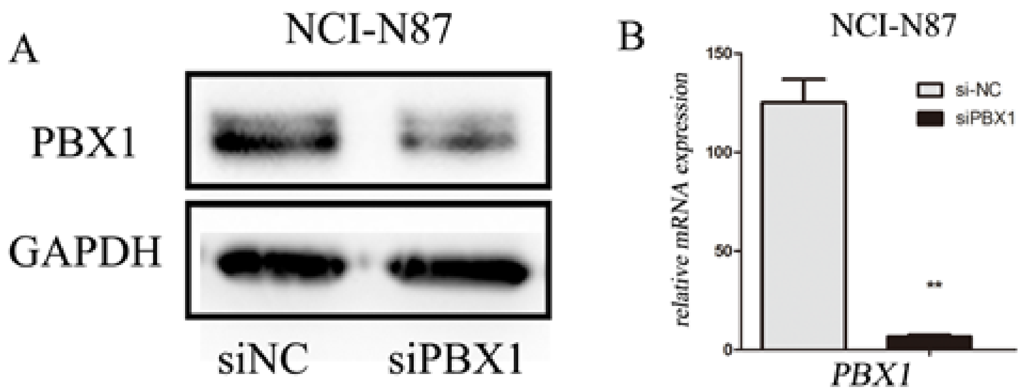

Supplementary Figure 1: The expression of PBX1 in NCI-N87 cells transfected with siNC or siPBX1. A, Protein expression of PBX1 in NCI-N87 cells transfected with siNC or siPBX1. B, mRNA levels of *PBX1* in NCI-N87 cells transfected with siNC or siPBX1. Data are represented as means ± SD of five independent experiments (***p* < 0.01).

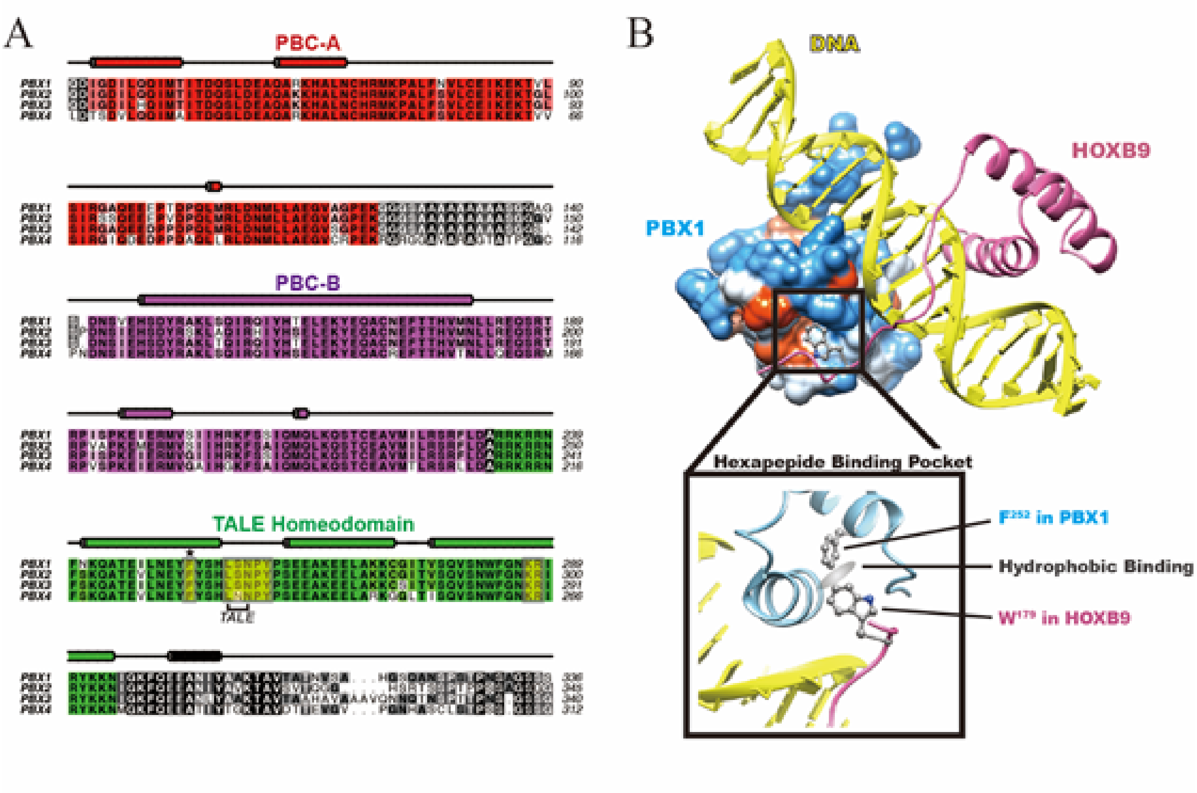

Supplementary Figure 2: Sequence alignment and structural studies of PBX1. A, The PBX family contains three conserved domains, PBC-A (red), PBC-B (purple) and TALE homeodomain (green). Residues of the hexapeptide binding pocket are represented in light green. A star indicates Phe^{252}. The predicted secondary structure is shown at the top and colored in accordance with aligned residues. B, A simulation model of the PBX1-HOXB9-DNA complex based on a crystal structure model of the HOXA9-PBX1-DNA complex (PDB: 1PUF). PBX1 is shown in a surface model (blue=acidic amino acids, red=basic amino acids, white=hydrophobic amino acids) and HOXB9 and DNA are represented as a ribbon model. The hexapeptide binding pocket of PBX1 is magnified and depicted as a stick and ribbon model.

For Supplementary Tables see in Supplementary Files.
