# Supplementary material for: A hydrophobic residue in the TALE homeodomain of PBX1 promotes epithelial-to-mesenchymal transition of gastric carcinoma
Source: Oncotarget. 2017 Apr 27;8(29):46818–33. doi: 10.18632/oncotarget.17473 (PMC5564525; doi:10.18632/oncotarget.17473)
Supplement: Supplementary file 2 [file oncotarget-08-46818-s002.docx]

**Supplementary Table 1**: **Primers for real-time PCR**.

| Name | Forward primer | Reverse primer |
| --- | --- | --- |
| *HOXB9* | GCCCGAGTACAGTTTGGAAA | TCTTTGTCCTCGCTTCCTTC |
| *PBX1* | AGGAAGCAGGACATTGGAGA | AGGCTTCATTCTGTGGCAGT |
| *TGF-β* | CAATTCCTGGCGATACCTCA | CTAAGGCGAAAGCCCTCAAT |
| *NRG2* | CGGCCCAAGTTGAAGAAGAT | AGCTCCTTGCCATCCTTGAA |
| *bFGF* | TGTGCTAACCGTTACCTGGC | TGGTGTATTTCCTTGACCGG |
| *VEGF* | GGCCTCCGAAACCATGAACT | TCGTGATGATTCTGCCCTCC |
| *MMP9* | AGACCTGGGCAGATTCCAAAC | CGGCAAGTCTTCCGAGTAGT |
| *GAPDH* | AGCCACATCGCTCAGACAC | GCCCAATACGACCAAATCC |

**Supplementary Table 2**: **Accession numbers of PBX proteins for sequence alignments**.

| **Name** | **Accession number** |
| --- | --- |
| PBX1 | NP_002576 |
| PBX2 | NP_002577 |
| PBX3 | NP_006186 |
| PBX4 | NP_079521 |

**Supplementary Table 3**: **PCR primers for vector construction and point mutagenesis**.

| Name | Forward primer | Reverse primer |
| --- | --- | --- |
| *(WT)* | CCGGAATTCGCCACCATGCATCACCATCACCATCACGACGAGCAGCCCAGGCTGATG | CCGGGATCCTCAGTTGGAGGTATCAGAGTGAACACTG |
| *PBX1*  *(Δ140)* | CCGGAATTCGCCACCATGCATCACCATCACCATCACTCAGACAACTCAGTGGAGCATTCAGATTACAG | CCGGGATCCTCAGTTGGAGGTATCAGAGTGAACACTG |
| *(Mut1)1* | CCGGAATTCGCCACCATGCATCACCATCACCATCACGACGAGCAGCCCAGGCTGATG | GGTTGCTGAGATGGGAATAAGCATATTCATTCAGGATTTCTGTCGCTTGCTTGTTG |
| *(Mut1)2* | CAGAAATCCTGAATGAATATGCTTATTCCCATCTCAGCAACCCTTACC | CCGGGATCCTCAGTTGGAGGTATCAGAGTGAACACTG |
| *(Mut2)1* | CCGGAATTCGCCACCATGCATCACCATCACCATCACGACGAGCAGCCCAGGCTGATG | CAGAAATCCTGAATGAATATCGATATTCCCATCTCAGCAACCCTTACC |
| *(Mut2)2* | CAGAAATCCTGAATGAATATCGATATTCCCATCTCAGCAACCCTTACC | CCGGGATCCTCAGTTGGAGGTATCAGAGTGAACACTG |

**Supplementary Table 4**: **Summary of PBX1 mutations and copy number variations (CNVs) identified in gastric carcinomas**.

| Position (AA) | Mutation | Mutation type | Mutation ID |
| --- | --- | --- | --- |
| 126 | S126L | Substitution - Missense | COSM4024698 |
| 133 | A133A | Substitution - coding silent | COSM4024699 |
| 183 | L183M | Substitution - Missense | COSM4024700 |
| 233 | A233V | Substitution - Missense | COSM40246701 |
| 260 | Y260C | Substitution - Missense | COSM4024702 |
| 402 | N402D | Substitution - Missense | COSM4024703 |

| CNV ID | CNV Type | Copy Number | Average Ploidy |
| --- | --- | --- | --- |
| 4331394 | Gain | 9 | 5.02 |
| 4334135 | Gain | 9 | 3.65 |
| 4422638 | Gain | 10 | 4.62 |
| 4461039 | Gain | 21 | 1.99 |
| 4461040 | Gain | 17 | 1.99 |

**Supplementary Table 5: Sequences of shPBX1 and siPBX1**

| Name | Sequence |
| --- | --- |
| shPBX1#1 | CAGGTTCAGACAACTCAGTGG |
| shPBX1#2 | CAGGTTCAGACAACTCAGTGG |
| siPBX1 | UCCUGCGUUCCCGAUUUCUTT AGAAAUCGGGAACGCAGGAUC |
